# Supplementary material for: Talent management of international nurses in healthcare settings: A systematic review
Source: PLoS One. 2023 Nov 6;18(11):e0293828. doi: 10.1371/journal.pone.0293828 (PMC10627454; doi:10.1371/journal.pone.0293828)
Supplement: S1 Checklist — (DOC) [file pone.0293828.s001.doc]

| **Section/topic** | **#** | **Checklist item** | **Location(s)** |
| --- | --- | --- | --- |
|  |  |  | **Reported** |
|  |  |  |  |
| **INFORMATION SOURCES** | **AND** | **METHODS** |  |
| Database name | 1 | Name each individual database searched, stating the platform for each. | P. 7, 8 |
| Multi-database searching | 2 | If databases were searched simultaneously on a single platform, state the name of the platform, listing all of the databases searched. | P. 7, 8, 9, 11 |
|  |  |  |  |
| Study registries | 3 | List any study registries searched. |  |
| Online resources and |  | Describe any online or print source purposefully searched or browsed (e.g., tables of contents, print conference proceedings, web sites), |  |
| browsing | 4 | and how this was done. | P. 7, 8, 9 |
|  |  | Indicate whether cited references or citing references were examined, and describe any methods used for locating cited/citing |  |
| Citation searching | 5 | references (e.g., browsing reference lists, using a citation index, setting up email alerts for references citing included studies). |  |
| Contacts | 6 | Indicate whether additional studies or data were sought by contacting authors, experts, manufacturers, or others. | P. 10 |
|  |  |  |  |
| Other methods | 7 | Describe any additional information sources or search methods used. |  |
| **SEARCH STRATEGIES** |  |  |  |
| Full search strategies | 8 | Include the search strategies for each database and information source, copied and pasted exactly as run. | P. 8, 9, 10 |
|  |  | Specify that no limits were used, or describe any limits or restrictions applied to a search (e.g., date or time period, language, study |  |
| Limits and restrictions | 9 | design) and provide justification for their use. | P. 10, 11  Figure 1 |
| Search filters | 10 | Indicate whether published search filters were used (as originally designed or modified), and if so, cite the filter(s) used. | P. 10, 11 |
|  |  | Indicate when search strategies from other literature reviews were adapted or reused for a substantive part or all of the search, citing |  |
| Prior work | 11 | the previous review(s). | P. 7  Protocol Paper (Ryan et al., 2022) |
| Updates | 12 | Report the methods used to update the search(es) (e.g., rerunning searches, email alerts). | P. 10 |
| Dates of searches | 13 | For each search strategy, provide the date when the last search occurred. | P. 10 Table 1 |
| **PEER REVIEW** |  |  |  |
| Peer review | 14 | Describe any search peer review process. | P. 11, 12, 15 |
| **MANAGING RECORDS** |  |  |  |
| Total Records | 15 | Document the total number of records identified from each database and other information sources. | P. 8 Table 1 |
| Deduplication | 16 | Describe the processes and any software used to deduplicate records from multiple database searches and other information sources. | P. 12, 13, 14, Figure 1 |

PRISMA-S: An Extension to the PRISMA Statement for Reporting Literature Searches in Systematic Reviews Rethlefsen ML, Kirtley S, Waffenschmidt S, Ayala AP, Moher D, Page MJ, Koffel JB, PRISMA-S Group. Last updated February 27, 2020.
