# Supplementary material for: Talent management of international nurses in healthcare settings: A systematic review
Source: PLoS One. 2023 Nov 6;18(11):e0293828. doi: 10.1371/journal.pone.0293828 (PMC10627454; doi:10.1371/journal.pone.0293828)
Supplement: Attachment — Submitted filename: Response to Reviewers.doc [file pone.0293828.s003.doc]

**Response Letter to Paper Reviewers: Ref PONE-D-23-21946**

Dear Editor,

At the outset we would like to thank you and the reviewers for taking the time to read our manuscript titled “Talent Management of International Nurses in Healthcare Settings: A Systematic Review” (PONE-D-23-21946) and for the detailed comments provided, which has allowed us to improve the overall quality of our manuscript. We have worked through each of the comments provided and have provided a detailed treaty of our response to each of these comments and the actions taken, as outlined in the table below. Based on the suggested changes, the paper’s focus and contribution has been significantly strengthened, adding important insight to this under-researched area.

For ease of reference, we have marked our changes in red throughout our revised manuscript and in the table below we provide each of the reviewers’ comments, our subsequent response, as the corresponding page number in our revised manuscript.

We look forward to hearing from you in due course.

Yours sincerely,

The Authors

| Sr No. | **Reviewer 1 Comments** | **Authors’ Response** | **Reference Pg No.** |
| --- | --- | --- | --- |
| 1 | In the method, it is not clear how the quality assessment of the included articles was made. | To start, we would like to take this opportunity to thank you for taking the time to review our manuscript. We have actioned each of your recommendations and provide details of the actions taken hereafter.  We appreciate your guidance on this point. We have added a more detailed outline on how the quality assessment of the included articles was carried out. | 17, 18 |
| 2 | Has the review been registered with the Open Science Framework (OSF) or PROSPERO? | Thank you for your point. The review has not been registered with PROSPERO. However, the protocol paper of this study was previously published in PLOS One and is provided as additional information, ref:  [Ryan, N. F., Berkery, E., O’Malley, B., O’Donnell, C., & Purtill, H. (2022). An evidenced-based approach to understanding and informing talent management practices for internationally trained nurses in healthcare: A systematic review protocol. Plos one,](https://journals.plos.org/plosone/article?id=10.1371/journal.pone.0278048)  [17(12), e0278048.](https://journals.plos.org/plosone/article?id=10.1371/journal.pone.0278048) | 7, 8 |
| 3 | It is not clear how the thematic analysis was carried out. In the results there is no description of the main themes (there is no analysis of the attached table), they are only presented in the discussion. | We appreciate your guidance on this point. We have added more details on the thematic analysis. We have included our thematic review methodology, and, in this section, we also outline and explain each of the main themes and how these themes were arrived at. We hope this provides full clarity to the readers. | 14-16 |
| 4 | The limitations of the review are not presented. | We recognise your point here and we apologies for this omission in our original submission. We have now included a limitations section in our revised draft. | 19 |

Reviewer II

| Sr No. | Reviewer’s Comments | Authors’ Response | Reference Pg No. |
| --- | --- | --- | --- |
| 1 | Why only 2012 to 2022 years were selected? Why not earlier? | Thank you for your point here, while Talent Management has been researched extensively in the private sector since the late 1990s, it has only gained prominence in the public sector from 2010 onwards, as outlined by Kravariti and Johnston (2020)- see [Foteini Kravariti & Karen Johnston (2020) Talent management: a critical literature review and research agenda for public sector human resource management, Public Management Review, 22:1, 75-95, DOI: 10.1080/14719037.2019.1638439](https://www.tandfonline.com/doi/full/10.1080/14719037.2019.1638439?casa_token=bbQzd7hajyEAAAAA%3AJypK6lhBLJ1NBaMgrCyWbR4XV-ngIDFMj8hlC0CSt5cfRqiqX28JDGDqEXO7uO2J2HbqIHnUCYwp)  Based on this the research team agreed to take the commonly used 10-year snapshot of research published between 2012 to 2022. |  |
| 2 | Also restricting to English language may also limit the review's generalization. This must be discussed as a limitation. | We appreciate your comment here. For the robustness of the systematic literature review, it is essential to have strict criteria for the inclusion and exclusion of the literature. This is why the authors decided to include the studies in English language only. Since all authors in the team are only English language speakers, these practical restraints based on resources available informed this criterion of inclusion was decided. Also, recent studies have shown minimal effect based on this criteria (Dobrescu, et al., 2021). As advised, we have included this point in the limitations section of the paper. | 11, 19 |
| 3 | Search terms on training, language and cultural competence, language barrier and economic status or financial reasons, support etc are not included and they have come as possible domains or themes in the end. | Thank you for your comment. The search terms used in this paper were taken from the protocol paper of this study published in PLOS One (Ryan et al., 2022). These search terms were discussed between the authors, followed by a consultation with the Glucksman Library at the University of Limerick. The search terms were discussed in depth with the librarians (who specialise in systematic literature reviews) and their advice was sought. The final search terms and search strings were decided after this consultative process. | 8 |
| 4 | In this topic grey literature may be important and why is it excluded may be explained in methods or in discussion? | We appreciate your guidance on this point. As the systematic literature review was designed to provide evidence for the development of effective talent management practices, the authors decided to include only academic studies that are peer-reviewed which they believe will provide a solid evidence-base in this area of research. We have now discussed this important point in the limitations. | 19 |
| 5 | Sensitivity analysis with covid onset/ peak as a time point would also be useful post hoc analysis. | We recognise your point and we have added this as a future research direction.  In our introduction we acknowledge the unforeseen pressure on global healthcare systems due to the onset of Covid-19, and now spotlight this as an avenue for future research. | 35 |
| 6 | How did the authors decide on thematic coding of 6 categories? How did these themes emerge? Thematic coding by one reviewer can be biased. How did the other reviewers contribute to the coding? Did they concur fully or were there any differences? | Thank you raising this point. The decisions about thematic coding were made by two authors in consultation with the other authors in the team. These decisions were reviewed in later stages as well and there were no disagreements. The details of this process are added to the methodology. | 14, 15, 16 |
| 7 | Risk of bias is also for individual study included in the study assessing for their quality and robustness of the results. This section needs rewriting and reporting on quality of the included studies. | Thank you for this important comment. The risk of bias and quality assessment of included studies is now added to the methodology in more detail. | 17-18 |
| 8 | Emigration to some countries is easier for people from specific countries and that may show up as preferences but while really be only convenience and necessity. | We appreciate your comment here. We have carefully considered the different aspects of talent management as being the main focus of our paper. This is why we deliberately excluded many studies that only considered the logistics of migration, migration motivations, registration processes, etc. and did not explicitly focus on the talent management of international nurses in healthcare settings.  Some of the excluded studies demonstrate the motivation of international nurses to work in other countries for better working conditions and improved lifestyle; thereby showing their preferences, however, this does not directly address ‘talent management of international nurses in healthcare settings’ and is not the emphasis of our paper. |  |
| 9 | With increasing risk of violence against heath workers and majority of nursing workforce being women, was there any themes on violence at workplace, harassment at workplace and harassment outside workplace be discussed in any of the include studies? | We appreciate your comment here. The thematic code of ‘Racism and Discrimination’ was added as an important aspect of the thematic analysis. The literature that was reviewed under this theme showed different experiences and dimensions of inequalities, exploitation and prejudice experienced by international nurses in their respective environments. However, these inequalities and exploitation did not necessarily demonstrate violence, therefore, the terms of racism and discrimination were considered more dominant in the reviewed studies. It was interesting to note that often the racism and discrimination experienced by international nurses was discreet in nature; therefore, it is discussed as microaggressions in the reviewed literature. | 15 |
| 10 | To refer to chapter and make changes as per recommendations and especially to correct section on risk of bias and to report same as per recommendations. | Thank you for sharing the reference link of the suggested chapter from Cochrane handbook of systematic literature reviews. We have made changes in accordance with the guidelines on this link. | 17, 18 |
| 11 | Articles excluded after full text review may be mentioned as a table with reasons for exclusion. | We recognise your point. As additional information, a table with articles excluded after full-article screening is provided along with reasons for exclusion. | Attached separately |
